# Incentives for mothers, health workers and “boda–boda” riders to improve community-based referral process and deliveries in the rural community: a case of Busoga Region in Uganda

**DOI:** 10.1186/s12978-022-01334-9

**Published:** 2022-01-28

**Authors:** Kharim Mwebaza Muluya, John Francis Mugisha, Peter Kithuka, Kenneth Rucha Kibaara, David Gangu Muwanguzi, Otieno George Ochieng, Andre Yitambe, Irene Wananda

**Affiliations:** 1 School of Public Health, Department of Health Management and Informatics, Kenyatta University, Nairobi, Kenya; 2 Cavendish University Uganda, Kampala, Uganda; 3 Iganga District Local Government, Iganga, Uganda; 4 Kamuli District Local Government, Kamuli, Uganda

**Keywords:** Incentives, Health workers, Boda–boda riders, Community-based referrals, Health facility-based deliveries

## Abstract

**Background:**

This study set out to investigate how incentives for mothers, health workers and boda–boda riders can improve the community-based referral process and deliveries in the rural community of Busoga region in Uganda. Both the monetary and non-monetary incentives have been instrumental in the improvement of deliveries at health centres.

**Methods:**

The study was a 2 arm cluster non-randomized control trial study design; with intervention and control groups of mothers, health workers and boba–boda (commercial motor-cycle) riders from selected health centres and communities in Busoga region. Among the study interventions was the provision of incentives to mothers, health workers (midwives and VHTs) and boda–boda riders for a duration of 6 months. Monetary and non-monetary incentives were applied in this study, namely; provision of training, training allowances, refreshments during the training, payment of transport fares by mothers to boda–boda riders, free telephone calls through establishment of a pre-paid Closed Caller User Group (CUG) and provision of bonus airtime to all registered CUG participants and rewards to best performers. The study used a mixed methods design. Descriptive statistical analysis was computed using STATA version 14 for the quantitative data and thematic analysis for qualitative data.

**Results:**

Findings revealed that incentives improved community-based referrals and health facility deliveries in the rural community of Busoga. The proportion of mothers who delivered from health centres and used boda–boda transport were 70.5% in the intervention arm and only 51.2% in the control arm. Of the mothers who delivered from the health centres, majority (69.4%) were transported by trained boda–boda riders while only 30.6% were transported by un-trained boda–boda riders. And of the mothers transported by the boda boda riders, 21.3% in the intervention arm reported that the riders responded to their calls within 20 min, an improvement from 4.3% before the intervention. Mothers who were responded to between 21–30 min increased from 31.4% to 69.6% in the intervention arm while in the control arm, it only increased from 37.1% to a dismal 40.3%. Interestingly, as the time interval increased, the number of boda–boda riders who delayed to respond to mothers’ calls reduced. In the intervention arm, only 6.2% of the mothers stated that boda–boda riders took as many as 31–60 min’ time interval to respond to their calls in post intervention compared to a whopping 54.9% in the pre intervention time. There was little change in the control arm from 53.2% in the pre intervention to 41.2% in the post intervention.

**Conclusion:**

Incentives along the maternal health chain are key and the initiative of incentivising the categories of stakeholders (mothers, midwives, the VHTs and the boda–boda riders) has demonstrated that partnerships are very critical in achieving better maternal outcomes (health facility-based deliveries) as a result of proper referral processes.

## Plain English Summary

Maternal and child health has become a priority area of focus among health policy makers and planners. Several interventions are being tried in an effort to increase deliveries in health facilities in order to realise better health outcomes. This study focused on how incentives for mothers, health workers and boda–boda riders can improve the community-based referral process and deliveries in the rural community of Busoga region in Uganda.

Two groups of boda–boda riders, mothers and health workers were followed up. One group was given incentives while the other was not. The incentives included training and training allowances, refreshments during training, payment of transport fares by mothers to boda–boda riders, free telephone calls and provision of bonus airtime to all registered CUG participants and rewards to best performers.

The results of this study showed that incentives help to boost deliveries from health facilities hence improving maternal outcomes. In intervention group, 70.5% of mothers who deliveries from health facilities were transported by boda–boda riders whereas this was only 51.2% in the control group. The response time of boda–boda risers to calls for transport by expecting mothers improved tremendously in the intervention group when compared to the control group. All the targeted parties—boda–boda riders, mothers, midwives and Village Health Teams were more active and motivated to work towards improving maternal health of the mothers.

## Introduction

Maternal and child health continues to be a largely overlooked aspect of health care system leading to major risks associated with pregnancy and child birth [9]. This could be associated with lack of interest in the information and interventions provided to the mothers [7]. Further, the deteriorating health of mothers and children has affected the national and global economy [5, 9]. Most times, mothers and children are hospitalised with limited time for productivity of both patients and the attendants. Many interventions like training of health workers and health educating of mothers have been tried but with little improvement in the maternal results. Perhaps these interventions required accompaniment of incentives to fully attract the involvement of mothers and other key stakeholders in the struggle for the improvement of maternal and child health.

Both monetary and non-monetary incentives have been instrumental in the improvement of deliveries at health centres. According to Ekirapa-Kiracho et al*.* [2], the voucher system as an incentive was introduced in Eastern Uganda where community motorcyclists (boda boda riders) accepted it in exchange for transport services for mothers to health centres during antenatal care (ANC), delivery and postnatal care (PNC). Initially, safe deliveries at health centres improved from 200 to over 500 deliveries per month in the intervention arm. In Malawi according to Akker et al*.* [1], non-monetary incentives which included soap, baby blanket and traditional baby wrap increased deliveries by 87% especially in the rural areas. According to Ir et al*.* [4], in Cambodia application of results—based financing contributed to the improvement in deliveries in health centres from 19 to 57% after 5 years.

Most of the studies which involved training of different stakeholders to improve maternal indicators did not consider much of the monetary incentives. The main focus of the studies was to provide knowledge as a non-monetary incentive though monetary incentives in form of allowances were later discovered to be vital. The Hunger Project [3] in Ghana in partnership with the Ghana Health Service (GHS) trained Community Health Nurses (CHN) as Midwife Assistants in form of workshops, mentorships and coaching for them to have enough information to give mothers, to be able to record properly in registers and to report in time. The health workers were pleased but the information received did not fully increase deliveries in the health centres. This was not different from the eastern study by Namazzi et al*.* [6] on training of Community Health Extension Workers (CHEWs) in assessing the danger signs in babies. Unfortunately, in all these studies transporters of mothers to health centres/hospitals were not involved in the education sessions. For effective uptake of the training intervention, incentives to mothers, boda–boda riders and health workers were important.

### Goal and objectives

The goal of this study was to establish the role of incentives to the mothers, health workers and boda–boda riders in improving community-based referrals and health facility-based deliveries in Busoga Region of Eastern Uganda. More specifically, the study aimed to achieve the following objectives:

1. To establish the role of incentives in improving deliveries in health units in Busoga region;
2. To examine the influence of incentives on the boda–boda riders’ responsiveness to the call by mothers for referral transport;
3. To find out the effect of time interval on deliveries at health facilities.

The overall justification of this study was based on the need to establish the role that monetary and non-monetary incentives play in improving maternal referrals and discovery of how raising awareness and interest through creating a collaborative partnership between mothers, boda–boda riders and the health workers would increase maternal referrals and deliveries in health facilities hence improving maternal health.

## Methods and materials

### Study design

The study was a 2 arm cluster non-randomized control trial study design; with an intervention group and a control group from selected health centres and communities. The study interventions involved providing incentives to mothers, health workers (midwives and VHTs) and boda–boda riders and this was done in a period of 6 months.

### Study setting

The study was carried out in the rural districts of Busoga in Uganda. Local level participants came from the districts of Bugiri and Iganga where study projects were conducted in selected Sub-Counties that made up the intervention and control arms. These sub counties included; Nambale, Nabitende, Nawandala in Iganga district and Budaya Sub County in Bugiri district for the intervention arm while Nawaningi, Ibulanku and Makuutu in Iganga district and Nabukalu in Bugiri district formed the control arm. The intervention health facilities included; Bugono health centre IV, Kasambika health centre III, Nambale health centre III, Nawandala health centre III, and Namusiisi health centre III in Iganga district and Mayuge health centre III in Bugiri. While the control arm included; Makuutu health centre III, Bunyiiro health centre III, Busesa health centre IV in Iganga district and Nabukalu health centre III in Bugiri district.

The reason for choice of study area was that motorcycle ambulances had once been operating under a funded project and had not been sustained. Therefore the study aimed at testing a local initiative that is more sustainable since it used locally available motorcycle (boda–boda) groups mobilised with the aim of referring mothers for further management at the different levels of health centres.

### Study participants

The participants in Table 1 were boda–boda riders, health workers (midwives and VHTs) and mothers. These were the primary target beneficiaries for the incentives and focus was based on these categories for this presentation in the intervention and control arms. Meanwhile, in the control arm, the study participants did not benefit from the incentives.

**Table 1:** Study participants in both intervention and control arms based on sub-counties

Categories of participants	Total number of participants		Study sub counties	
	Intervention arm	Control arm	Intervention arm	Control arm
Boda–boda riders	100	92	Budaya, Nawandala, Nabitende and Nambale	Nabukalu, Nawaningi, Makuutu and Ibulanku
Midwives	18	18		
Mothers	255	248		
Village Health Team Members	8	10		
Total	381	368		

### Recruitment of participants

The mothers were recruited from the antenatal register based on their willingness and meeting the inclusion criteria and in the selected study villages and sub counties. Mothers in their third trimester were recruited purposely to determine the impact of the incentives. Mothers were recruited in both the intervention and control arms.

The boda–boda riders were recruited basing on their operating stage (work station), activeness and willingness to take part in the study and be trained. Boda–boda riders who were from boda–boda stations within the study sub counties who had no registered crime with police were selected.

The midwives from the selected health facilities were recruited basing on their expressed interest and experience in research. They must have worked with the health facility for over 6 months. The VHTs were recruited purposively based on their presence in the selected sub counties together with their willingness to take part in the study. Also, very active VHTs were considered for this study.

Consent was sought from all the participants (midwives, boda–boda riders, VHTs and mothers) before they were initiated into the study.

### Sampling techniques of participants

For this study, purposive and random sampling techniques were employed. Study participants were identified from different data sources such as delivery register, ANC register and PNC registers in the selected health centres, VHTs registers at the district health office and boda–boda riders’ register at the village, sub county or district levels. Random methods were used to select from a given register to give each qualifying member a chance.

### Training of stakeholders and other incentives

One of the cross-cutting interventions was training. Four training sessions were conducted in the selected four sub counties for the intervention group for the boda–boda riders and health workers. The training lasted five days. Mothers on the other hand were trained from the health centres during the ANC visits. Participants in the training were facilitated and compensated for the days in training since some of the riders did not own motorcycles and were supposed to deposit their commissions to the motorcycle owners. Sessions were conducted for a relatively short period of time (9 am–2 pm) daily to allow the riders continue with their daily work after the training since there was demand for their services in the community. The VHTs and midwives were also facilitated with allowances in the training.

Training sessions of mothers at health centres took a shorter time of around 1–2 h. These were mothers in their third trimester. As an incentive for the training, mothers were given only refreshments on every ANC visit.

### The closed caller user group and the bonus airtime

Another intervention was use of the closed caller user groups (CUG). Participants who benefited from this were the mothers, boda–boda riders, VHTs and midwives. Participants in the control arm did not benefit from the CUG. On consent, participants in the intervention arm were registered by the MTN telecommunication company to benefit from the free communication calls between all the members in the group.

As another strategy, a bonus airtime of 10,000 Uganda shillings was given to each member of the group for communicating to other members who were not in the group during times of emergency. This was in a situation where the boda–boda rider was far and would use the bonus airtime to call another boda–boda rider at the stage to stand in for him regardless of whether he was in the CUG or not.

### Transport fares of boda–boda services for mothers

In order for mothers to fully benefit from the boda–boda services, they were continuously encouraged to save money in the savings boxes for a sustainable motivation of boda–boda riders, as an intervention. This money was to pay for mothers’ transport fares for boda–boda services. It motivated the boda–boda riders because they were assured of their payments for the services. There was increased access and quality of health care services in local communities.

The terms of payment for boda–boda transport services were mutually agreed upon between the mothers and the boda–boda riders in accordance with the distance travelled to reach the health facility. The study commenced with a baseline survey in both groups so as to get statistics for comparison after the intervention. This was important for determining the extent of the changes brought by the intervention.

### Research instruments

*Questionnaires*: We had baseline and exit interview questionnaires that were filled at the health facility for the mothers who used boda–boda transport in to the health centres.

*Interview guide*: Data was also collected using the key informant and in-depth interview guides for the boda–boda riders, mothers, VHTs and midwives in selected health facilities of the intervention and control arms.

*Focus group discussions (FGDs)*: This was done at baseline as an entry point and later on when executing the study. It consisted of different stakeholders in the study.

*Document review checklist*: this was designed to capture data from the secondary source. These included; delivery registers, ANC registers and PNC registers.

### Data analysis

Data analysis of descriptive statistics was computed using STATA version 14 for the quantitative data. Paired t-tests of independence were used to determine the statistical significance of the different variables with *p*-value set at 0.05 and confidence interval at 95%.

In order for the study to be informative, difference-in-difference (DID) framework was used. The simplest form of the DID design is a special case in which there are only two groups observed in two time periods. DID was used to determine the change effect based on the average value and its statistical significance.

Atlas Ti version 7 was used for qualitative analysis. It involved re-reading the interview transcripts to identify themes and sub themes that emerged from the respondents’ answers during the FGDs, Key Informant Interviews (KIIs) and In-depth Interviews (IDIs). The arrangement for analysis was based on the topics and questions formulated for the interviews in order to synthesize the answers to the proposed questions.

## Results

The results are presented in terms of the intervention strategies that were applied to different participants in the intervention arm; and how they affected the maternal health outcomes. These included: the training component, closed caller group, effect of the bonus airtime and other general incentives in form of allowances to training participants, payment for services as well as non-monetary incentives such as recognition and rewarding of best performers during meetings. The analysis shows how these different intervention strategies impacted the maternal health outcomes.

### Training incentives and its impact

Training incentives included; training allowances to midwives, boda–boda riders and VHTs. Also, mothers were given refreshments at the health centre during ANC visits. The proportion of mothers who delivered from health centres and were transported by boda–boda transport was 70.5% in the intervention arm and only 51.2% in the control arm. And of these 70.5% of the mothers who delivered from the health centres, 69.4% were transported by trained boda–boda riders and only 30.6% of the mothers were transported by un-trained boda–boda riders. This gives a basis to suggest that incentivising and training of the boda–boda riders while linking them to the mothers had an impact on boosting deliveries in health facilities.

This is also supported by the qualitative information which was provided by the different stakeholders during and after the training. It appears that a mix of excitement, friendship creation and participatory learning were witnessed during and after the training. One of the participants (a boda–boda rider) noted;

“...I have learnt a lot from the training, for example the importance to take mothers to the health facility in time. Since you stated that communication was simplified for mothers to contact us, then we shall also respond very fast”

The health workers noted the need for team work and commitment by all those involved in the study would enable them to meet the needs of mothers who are in dire conditions of child birth.

The excited VHTs who participated in the training indicated that they should not only enjoy the allowances which were given to them but also emphasised that this training was very timely especially for the boda–boda riders.

One of the VHTs in the training room emphasised to the boda–boda riders that they should put into practice what was learned:

“...Knowledge building and sharing on understanding of MCH is now seen on your faces, especially you the boda–boda riders who now know the need to support the mothers by timely transporting them to the health centres for delivery. Let us be practical.”

### Transport fare payments by mothers to the boda–boda riders

Mothers were encouraged to save money in small boxes which were broken at the time they were experiencing labour pains. Boda–boda riders were able to respond to mothers’ calls very fast because they were very sure of their payments when they offered the transport services. As seen before, 70.5% of the mothers who delivered from health facilities were transported by the trained boda–boda riders.

One of the stage coordinators of the boda–boda riders indicated during an in-depth interview that in the past, he did not consider it urgent to provide transport to mothers in labour or mothers with birth related complications to the health centres. This is because he never had sufficient knowledge of the urgency and the labouring mothers did not have money to pay them on arrival at the health centres.

“...what we have learnt today is important and I am committed to … save mothers, even if it means taking them to the health facility on credit. However, since they are saving it will not be a challenge for them to pay us.”

### Free calls, bonus airtime and time interval taken for the boda–boda rider to arrive

The incentive for the CUG was the free calls and the bonus airtime. Participants who majorly benefited from the CUG included; mothers, health workers and boda–boda riders. The free calls and bonus airtime enjoyed in CUG contributed to the reduction in time boda–boda riders took to respond to mothers’ calls. The results are presented in Table 2 that follows:

**Table 2:** Communication by mothers for boda–boda transport

		Pre		Post			
Yes		Interv N = 255	Control N = 248	Interv N = 255	Control N = 248	DID	P-value
Time interval taken for the boda–boda rider to arrive when contacted by the mother (yes)	5–20 min	11 (4.3%)	18 (7.3%)	54 (21.3%)	41 (16.7%)	0.391	0.000
	21–30 min	80 (31.4%)	92 (37.1%)	177 (69.6%)	100 (40.3%)		
	31–60 min	140 (54.9%)	132 (53.2%)	16 (6.2%)	102 (41.2%)		
	60 + min	24 (9.4%)	6 (2.4%)	8 (2.9%)	5 (1.8%)		

The bonus airtime and free calls were important in the coordination of mothers’ transport to health centres. The mothers in the CUG could easily contact boda–boda riders for transport services because of free calls. Similarly, boda–boda riders were able to call back the mothers and health workers.

Of the mothers who were transported by the trained boda–boda riders to health centres for delivery, those who stated that boda–boda riders’ response to their calls took 5–20 min improved from 4.3% to 21.3% in the intervention arm. Those who were responded to between 21 and 30 min also improved from 31.4% to 69.6% in the intervention arm; this compared to a small increase from 37.1% to only 40.3% in the control arm. This shows how training of boda–boda risers impacted their response to mothers’ call for transport.

Actually as the time interval increased, the number of boda–boda riders who delayed to respond to mothers’ calls reduced. For instance, Table 2 above shows that in the post intervention in the intervention arm, only 6.2% of the mothers stated that boda–boda riders took 31–60 min to respond to their calls compared to 54.9% in the pre intervention. There was little change in the control arm from 53.2% in the pre-intervention to 41.2% in the post-intervention. Similarly, the number of boda–boda riders who took time to respond to mothers’ calls when contacted for transport reduced for the time interval of 60 min from 9.4% to 2.9% in the intervention arm as shown in Table 2.

According to the DID model of analysis, the intervention was impactful. The time interval boda–boda riders took to respond to mothers’ calls for transport kept on reducing. The average value for the time interval boda–boda riders took to arrive when contacted was 0.391. This was found to be statistically significant to the change effect as shown in Table 2 (p = 0.000). Therefore, the allowances and other incentives improved on the responses made by the boda–boda riders when contacted by mothers for transport to health centres and had effect on the time interval it took the riders to reach mothers and deliver them to health facilities for safe delivery.

### Influence of time intervals on deliveries at health centres

Regression analysis of the time interval boda–boda riders took to respond to mothers’ calls had a statistically significant influence on the deliveries at health centres. The results in Table 3 exhibit this:

**Table 3:** Time interval and its association with health facility-based deliveries

Predictive variable		Odds ratio (95%)	P-value
Category	Control	*1*	
	Intervention	1.173 (0.760, 4.501)	**0.001**
Time interval taken for the boda–boda rider to arrive when contacted by the mother	5–20 min	*1*	
	21–30 min	0.344 (0.189, 0.626)	**0.000**
	31–60 min	0.027 (0.007, 0.103)	**0.000**

The incentives to mothers, boda–boda riders and other stakeholders increased their motivation and improved deliveries in health centres. The motivation of these stakeholders reduced the time boda–boda riders took to respond on the mothers’ calls when contacted for transport to health centres. Results show that there was a statistically significant influence of time interval on health facility-based deliveries. Pregnant mothers who contacted boda–boda riders at the different intervals for transport to health centres in the intervention arm were 1.173 times more likely to deliver from health centres compared to those in the control arm (p = 0.001; CI = 0.760–4.501).

Considering the statistical significance of the time intervals, the 5–20 min interval which boda–boda riders took to arrive when contacted by mothers had no association with deliveries at health centres. The other time intervals (21–30 min and 31–60 min were statistically significantly influencing the deliveries in health centres (OR = 0.344; p = 0.000; CI = 0.189–0.626 and OR = 0.027; p = 0.000; CI = 0.007–0.103, respectively).

During an FGD in the intervention arm specifically in the pre-intervention phase, boda–boda riders were encouraged to be quick and respond whenever a mother contacts them. They were assured of immediate payments on delivery of mothers at the health centres. Mothers had saved money in the saving boxes.

“…Do not take much time to go to mothers whenever they call you. Just know, that is a hard moment especially when in labour. Mothers cannot walk at that time. These are our relatives, wives, daughters and sisters. Learn to rush to them.” One of the members of the group urged the colleagues.

### Rewards of best performers: non-monetary

Quarterly meetings in the different sub counties in the intervention arm were conducted. In these meetings, discussions were focussed on the improvement of community-based referrals and increased deliveries in health centres. Boda–boda riders who transported the biggest number of mothers were rewarded with helmets and recognition during meetings. The majority of the boda–boda riders appreciated the importance of the meeting.

“...In these meetings we are able to present our challenges we face in the transportation of mothers such that solutions are discussed here.” One of the boda–boda riders stated.

Another one said;

“…I’m not the best boda–boda rider pronounced in this meeting and this has given me the zeal to work hard such that next time I become one of the best riders in my sub county”.

Similarly, in addition to the routine allowances which were given to the different midwives for the recruitment and registration of mothers in the CUG, boda–boda riders gave a feedback of the best midwives who were cooperative. These were rewarded and recognised before the boda–boda riders in the meetings.

“...I did not know that by the humble interaction with the local boda–boda riders I was doing something appreciated by most of them. This has opened my mind and now I know the reason for the increasing number of deliveries at my health centre”. A midwife who was excited stated in one of the meetings.

## Discussion

Incentives are a key component of performance in this study. All the different categories of participants were motivated in one way or the other with incentives. The different participants were given incentives to take part in the study; these participants are the health workers, the boda–boda riders, and the pregnant women in their third trimester. This was not different from other studies. According to Ekirapa-Kiracho et al*.* [2] in the voucher system used as an incentive in eastern Uganda, like in the rural Busoga sub region study, community motorcyclists accepted to transport mothers to health centres during antenatal care (ANC), delivery and postnatal care (PNC). Further, in the Ekirapa-Kiracho et al*.* [2] study, safe deliveries at health centres improved from 200 to over 500 deliveries per month, and similarly, deliveries increased from 52% to 70.5% in the rural Busoga sub region study.

The introduction of the non-monetary incentives in the rural Busoga sub region study like it was in the Malawi study, resulted in the improvement in the deliveries at the health centres. In the rural Busoga sub region study, incentives included recognitions during meetings unlike in Malawi where soap, baby blanket and traditional baby wraps were distributed according to Akker et al*.* [1], but had similar results (improved deliveries). Non-monetary incentives in Busoga region study included; free calls and bonus airtime for boda–boda riders and mothers who were in the closed caller user group. Incentives were given for a period of 6 months and deliveries for mothers transported by boda–boda riders increased to 70.5% compared to 87% in the rural areas of Malawi for the 2 years as stated by Akker et al*.* [1]. The study has not tested the sustainability of having good results without the intended incentives to the different stakeholders.

Mothers were able to call members of the group even without airtime or credit on their phones. This made it easy to link the mothers to the VHTs, boda–boda riders and the health workers (midwives) in the health centres for participating sites and cheap to call the boda–boda riders to take the mothers to the health facilities without any delays. The free calls and bonus airtime led to improved communication time and response for taking the mothers to the health facilities. In situations where the boda–boda rider was far, a mother would use the bonus airtime to call another boda–boda rider at the stage regardless of whether he was in the caller group or not. This increased the number of health facility deliveries from 52% to 70.5% in the sub region.

While for the VHTs, referral was made easier since they were able to call the midwife in the study facility and give their quick assessment of the situation of the mother before she reaches the health centre. The midwife prepared depending on the information shared by the VHT who was referring the mother from the community. Timely and functional referral system for mothers in the community is important as it backs up antenatal care, labour and delivery at low and high level health facilities [8]. This was exhibited in the rural Busoga sub region study.

Like the voucher system in the Ekirapa-Kiracho et al*.* [2] study, the rural Busoga region study incorporated a savings system for the expectant mothers to put a minimum of 200 shillings in a “savings box” per day for almost 3 months before they give birth. This money was for the basic needs during delivery including transporting the mother to the health facility and motivated boda–boda riders to willingly transport mothers to health centres. This incentive is sustainable since majority of mothers are capable of saving in the boxes. Mothers, health workers and boda–boda riders having information and communication systems, their attitude towards community referrals changed and it was instrumental in improving some of the maternal health indicators. This is crucial in reducing the delays to seek quality healthcare services.

### Methodological considerations

Although we tried to reach all the members in the study population and several categories of stakeholders who might influence the intervention, we were unable to interview some key stakeholders such as a few opinion leaders. Another limitation of this study is that the views of those interviewed may not be generalised to the entire institutions they serve or work for. Further still the views of stakeholders may change with time. Since some of the incentives were monetary, its sustainability is still questionable. However, despite the inherent limitations, stakeholder analysis is a vital tool in informing the design of any form of health systems research or intervention.

## Conclusion

The incentives have proved to be an effective way to improve maternal and neonatal outcomes especially in rural communities in the less developed countries. Incentives along the maternal health chain are key and the initiative of incentivising the three categories of stakeholders (the health workers- midwives, the VHTs and the boda–boda riders) with the closed caller group for free calls, bonus airtime and an extra allowance for the health workers has demonstrated that partnership are very critical in achieving better results or maternal outcomes especially the health facility-based deliveries.

The incentives have the potential to increase the interest and commitment of boda–boda riders in transporting mothers to the health facilities as a priority to other customers since they feel obliged. This is a key component in addressing the challenges of complications for women who are at the verge of child birth.

It also addresses the other challenges of response time as it is clear that in developing countries most maternal and neonatal deaths can be prevented if there is a robust system to refer, transport and manage the delivery process especially for mothers with complications.

We recommend that Government should support riders through credit extension to buy and own motorcycles as those who were using hired ones had more challenges. All mothers in the region should be encouraged to start savings boxes during the first ANC visit in all health centres to gather enough money for transport fare and other necessities during time of delivery. Boda–boda riders should be branded for easy identification since they are all willingly transporting mothers to health centres to deliver.

## Data Availability

No further data and material can be shared without express permission of study participants.

## Abbreviations

ANC: Antenatal care
CHEW: Community health extension worker
CHN: Community health nurse
CI: Confidence interval
CUG: Caller user group
DID: Difference-in-difference
FGD: Focus group discussion
GHS: Ghana health service
IDI: Indepth interviews
KII: Key informant interviews
MoH: Ministry of Health
PNC: Postnatal care
VHTs: Village health teams
WHO: World Health Organisation
